# Fluorescence Intensity Normalisation: Correcting for Time Effects in Large-Scale Flow Cytometric Analysis

**DOI:** 10.1155/2009/476106

**Published:** 2009-11-17

**Authors:** Calliope A. Dendrou, Erik Fung, Laura Esposito, John A. Todd, Linda S. Wicker, Vincent Plagnol

**Affiliations:** Juvenile Diabetes Research Foundation/Wellcome Trust Diabetes and Inflammation Laboratory, Department of Medical Genetics, Cambridge Institute for Medical Research, University of Cambridge, Cambridge CB2 0XY, UK

## Abstract

A next step to interpret the findings generated by genome-wide association studies is to associate molecular quantitative traits with disease-associated alleles. To this end, researchers are linking disease risk alleles with gene expression quantitative trait loci (eQTL). However, gene expression at the mRNA level is only an intermediate trait and flow cytometry analysis can provide more downstream and biologically valuable protein level information in multiple cell subsets simultaneously using freshly obtained samples. Because the throughput of flow cytometry is currently limited, experiments may need to span over several weeks or months to obtain a sufficient sample size to demonstrate genetic association. Therefore, normalisation methods are needed to control for technical variability and compare flow cytometry data over an extended period of time. We show how the use of normalising fluorospheres improves the repeatability of a cell surface CD25-APC mean fluorescence intensity phenotype on CD4^+^ memory T cells. We investigate two types of normalising beads: broad spectrum and spectrum matched. Lastly, we propose two alternative normalisation procedures that are usable in the absence of normalising beads.

## 1. Introduction

Genome-wide association (GWA) studies have revolutionised the mapping of common genetic variants, mostly single nucleotide polymorphisms (SNPs), with susceptibility to a wide range of common, multifactorial disorders [1], in particular autoimmune diseases [2]. The next step to followup on these findings is the identification of the molecular effects of these genetic risk variants. A potential approach to achieve this goal is to associate these risk alleles, in sufficiently large cohorts, with quantitative molecular traits. This approach has been widely used in the context of gene expression mRNA analysis [3–6] but RNA is only an intermediate step and downstream protein level traits provide more valuable biological information. 

Multicolour flow cytometry analysis can provide rich protein level data simultaneously on different subsets of cells; this is of particular importance for post-GWA investigations as genetic heterogeneity identified in disease-associated regions can differentially affect various cell subsets. However, the throughput of current flow cytometry approaches, including data analysis and sample collection, is limited to a small number of samples per day or week, especially when fresh blood is required. As the identification of subtle molecular effects directed by common genetic variants may require the analysis of a relatively large number of samples, flow cytometry experiments may need to span over several months. Owing to the complexity of flow cytometry technology, various technical artifacts, including variability in reagents or measuring instruments, can create time-related biases. Consequently, normalisation procedures are necessary to enable the comparison of samples analysed at different dates.

Similar issues have been identified in the context of gene expression microarray analysis. For these analyses researchers typically take advantage of the large number of independent measurements (one per gene or probe), implicitly using the rank of a gene of interest as a summary statistic. Such techniques are not available for flow cytometry data, and therefore specific approaches are required.

With the motivation of understanding the molecular effects of type 1 diabetes (T1D) risk variants located in the IL2 receptor *α*-chain (IL-2RA/CD25) gene region [7], we quantified cell surface expression of CD25 on CD4^+^ T cells using flow cytometry [8]. We analysed 192 samples over a seven-month period, including 15 pairs of repeated individuals (with blood donations separated by at least three months) in order to assess measurement repeatability. We show how time-related biases affect the repeatability of a phenotype of interest, computed as a mean fluorescence intensity (MFI) in a population of CD4^+^ memory T cells. We used the repeatability level of this genetically controlled and stable phenotype as a proxy for technical variability of the flow cytometry measurements. We show how using fluorescent calibration beads to normalise the MFIs can control for day-to-day technical variability, generated by the flow cytometer, that could not be controlled for otherwise.

## 2. Results

### 2.1. Repeatability of CD25-APC Normalised Mean Fluorescent Intensity (MFI) Phenotype

**Figure 1:**
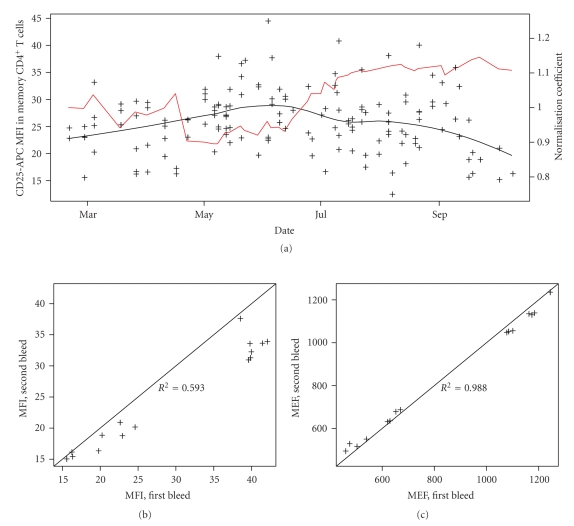
(a) Black crosses show nonnormalised MFIs in the CD4^+^ memory T cell population as a function of time. The back line was fitted line to these MFI values using a loess procedure. The red line shows the normalisation coefficient estimated from the beads. (b) Repeatability plots (*n* = 15 pairs) for MFIs of CD25-APC cell surface expression in the CD4^+^ memory T cell population. (c) Repeatability plots (*n* = 15 pairs) for CD25-APC MEF (normalised MFI) in the same cell population. For (b) and (c), each individual's blood donations were separated by at least 3 months.

Using multicolour flow cytometry analysis, we previously identified CD25 cell surface expression on CD4^+^ memory T cells to be associated with genetic variants in the CD25 gene region [8]. This phenotype is a MFI of anti-CD25 conjugated to APC in this cell population. To analyse this cell population the 192 samples were gated manually (using the software FlowJo, Tree Star, Inc.) to correct for interindividual and technical variability (see Figure S1 in supplementary material avaliable online at doi: 10.1155/2009/476106 for a description of gating procedure). Constant flow cytometer settings, pooling of different antibody batches prior to the start of the study, and strict protocol adherence were used to control for technical variability. Nevertheless, when analysing the distribution of this MFI phenotype across time, we observed significant time effects. Because this phenotype is correlated to *CD25* genotype, we restricted this analysis to 149 samples with an identical T1D susceptible *CD25* genotype at the main CD25 expression associated SNP [8]. However, time-associated trends remained significant even in this subgroup (*p* = 5 × 10^−4^ when regressing the MFI against a quadratic model for time, coded in number of days, see Figure 1(a)). These time effects are probably due to fluctuations in the flow cytometer that cannot be measured.

To better control for technical day-to-day variability of the flow cytometry measures, MFIs were converted to molecules of equivalent fluorochrome (MEF) using six peak calibration beads (Dakocytomation, see Methods). For each experimental day, the MFIs of the six peak calibration beads were measured using flow cytometer settings identical to the ones used for the analysed samples. Using the MFI to MEF correspondence provided by the manufacturer we fitted a linear model MEF = *α* × MFI and used this linear transformation for MFI normalisation. The efficiency of this procedure is illustrated by the improved repeatability of the MEF in contrast with the nonnormalised MFI (Figures 1(b) and 1(c)), thus demonstrating an improved control for day-to-day technical variability.

### 2.2. Background Subtraction Using Isotype Control

**Figure 2:**
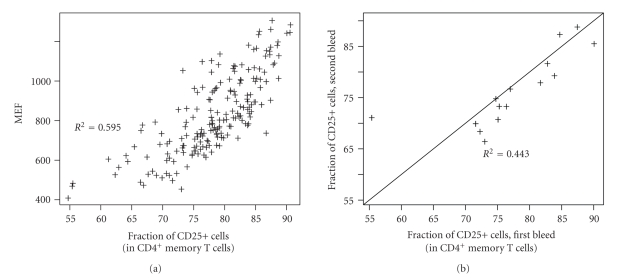
(a) Correlation between the fraction of CD25-positive cells in the CD4^+^ memory T cell population and the CD25-APC MEF in this population. (b) Repeatability (*n* = 15) of the estimated fraction of CD25-positive cells in the CD4^+^ memory T cell population obtained by background subtraction of the isotype control distribution.

Typical flow cytometry procedures to control for day-to-day technical variability use a fluorochrome-conjugated isotype control antibody to quantify the background, nonspecific, fluorescence intensity. Subtraction procedures are then applied to compare the background level with the observed intensity in order to estimate the fraction of positive cells, as defined by cells with a fluorescence level exceeding background [9]. In the example described here, measures obtained using background subtraction (either two-percent of background or maximum positive difference, see [9]) are less replicable (*R* ^2^ = 0.443) and correlations with the MEF phenotype are limited (*R* ^2^ = 0.59, see Figure 2).

These differences are consistent with the fact that the MFI and the fraction of CD25+positive cells provide different types of information. Therefore, these summary statistics require different normalisation approaches: one using normalisation beads, the other using an isotype control.

### 2.3. Broad Spectrum versus Spectrum Matched Beads

The calibration beads used in this study are broad spectrum beads, which means that the same set of beads can be used to normalise fluorochromes at different wavelengths (e.g., PE and APC using the same set of beads). Alternative normalisation tools use spectrum matched beads, that is, fluorescent beads whose light spectrum matches exactly the fluorochrome of interest, for example, APC. Such spectrum matched beads are required to standardise flow cytometry measurements across different laboratories or flow cytometers [10]. The fact that the data presented in this study were generated using a single flow cytometer (BD Biosciences LSRII) limits the complexity of MFI normalisation, thus justifying the use of broad spectrum beads.

**Figure 3:**
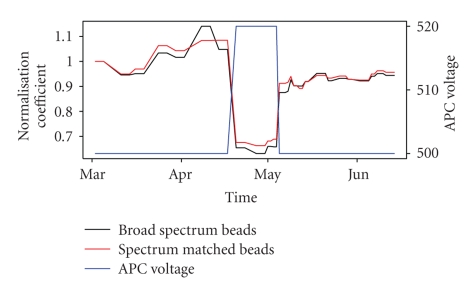
Variability across time of the normalisation coefficient for broad spectrum beads (black) and APC spectrum matched beads (red). The blue line shows the APC photomultiplier tube voltage setting used to measure the beads MFI.

**Figure 4:**
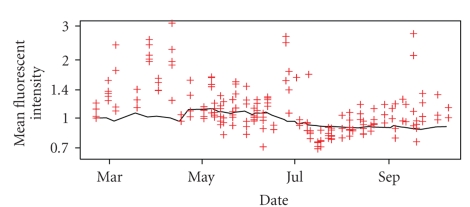
Variability across time of the isotype control MFIs (red crosses, one point per sample) and the normalising beads MFIs (black line, one point per experimental day). MFIs are scaled such that the value is equal to one for the first day, and a logarithmic scale is used for the *y*-axis.

**Figure 5:**
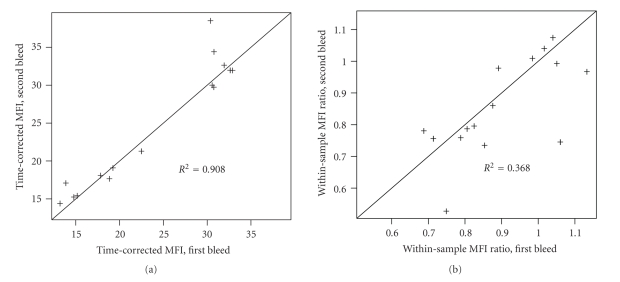
(a) Repeatability for the CD4^+^ memory T cell population CD25-APC MFI normalised using a multiplicative correction factor estimated by a regression analysis on the set of 149 samples with identical T1D susceptible genotype. (b) Repeatability for the CD4^+^ memory T cell population CD25-APC MFI divided by the CD25-APC MFI in the full CD4^+^ T cell gate for the same sample/analysed tube.

To better understand the impact of broad versus spectrum matched normalising beads, we analysed normalising beads from another dataset generated during the same time period using the same flow cytometer. For this additional dataset broad spectrum (Dakocytomation) and APC spectrum matched (BD Biosciences) were tested. For technical reasons, and also to better understand the effect of variability in photomultiplier tube voltage (controlling the light detection sensitivity), flow cytometer settings were not kept constant through time for these additional beads data. Indeed we observed that, as expected, the normalisation coefficient is strongly negatively correlated with the APC photomultiplier tube voltage (Figure 3). Note that, in contrast with the data in Figure 3, the APC voltage remained constant for all other data (Figures 1, 2, 4, and 5), and, therefore, differences in voltage settings explain the differences in MFI trends between Figures 1(a) and 3. We found very close agreement between broad spectrum and APC spectrum matched beads (Figure 3), hence justifying the use of broad spectrum beads if the analysis involves a single flow cytometer operated under a strictly adhered-to protocol.

### 2.4. Isotype Control Is Not Usable for MFI Normalisation

We then investigated whether MFIs obtained by measuring the isotype control fluorescence are usable for MFI normalisation, in contrast with the traditional use for background subtraction. Isotype controls are primarily used to provide information on nonspecific binding via Fc receptors present on the cells of interest. In our analyses, we attempted to block such Fc binding using mouse IgG immunoglobulin (Sigma-Aldrich Company), thereby making the isotype control primarily a measurement of the autofluorescence [11] of the cell population examined. We hypothesized that, owing to this nonspecificity, the biological donor-to-donor variability would have a more limited effect on isotype fluorescence, thus providing some information of technical variability.

In Figure 4, we show a comparison of the variability across time of the normalising beads and isotype control MFIs. We found that the variability of the isotype control MFIs greatly exceeds the variability obtained from normalising beads. A regression analysis using a quadratic model for time (coded as number of days) regressed against the average isotype MFI for each day explains only 18.4% of the variance of the isotype MFI values. The same regression for the normalising bead MFIs explains 64.8% of the measurement variance. The isotype MFIs also showed large variation across different donors analysed on the same day, suggesting that donor-to-donor differences in autofluorescence levels contribute to the isotype MFI variability. Moreover, for low MFI values in the range of the isotype control MFIs, the signal-to-noise ratio is low.

Overall, the isotype MFIs are highly variable and affected by donor-to-donor variability. In addition, the biological variability captured by the isotype control MFIs (average MFI less than 2) is not significant when analysing higher CD25 cell surface MFIs in the CD4^+^ memory T cell population (average MFI: 25). Thus, the biological donor-to-donor information captured by the isotype control is not relevant for normalising the MFIs of interest. Taken together, these results indicate that the isotype control is not usable for MFI normalisation.

### 2.5. Across-Sample Normalisation in the Absence of Calibration Beads

We then investigated alternative procedures allowing for the control of flow cytometry day-to-day technical variability in MFI measurements in the absence of calibration beads. First, we investigated whether we could use the 192 samples analysed to estimate the trend associated with technical variability, and use this estimate to correct for time-related biases. Because of CD25 genotype-phenotype correlations [8], we only included 149 individuals with identical T1D susceptible genotypes at the main CD25 expression associated SNP. We coded time as the number of days since the first bleed and regressed a quadratic model for time against the CD25-APC MFI estimated in the total CD4^+^ T cell population to generated predicted values *p*_*t*_. The multiplicative normalising factor was estimated as *α*_*t*_ = *p*_*t*_/*p*_*t*=0_. Applying this correcting factor to our main phenotype of interest (CD25-APC MFI in the CD4^+^ memory T cell population, Figure 5(a)) significantly improved the phenotype repeatability (*R* ^2^ = 0.91) and helped control for time-related biases.

### 2.6. Within-Sample Normalisation in the Absence of Calibration Beads

We then investigated a second procedure for MFI normalisation, a flow cytometry approach analogous to quantile normalisation for gene expression microarray data. In the context of microarray data, quantile normalisation takes advantage of a large number of independent data points (one point per gene or probe) to rank a gene of interest within the overall distribution of gene intensities. This procedure corrects at least partially for variability across independent microarray experiments. Flow cytometry analysis, on the other hand, does not provide large numbers of independent data points. However, some partially uncorrelated MFI measures are available when analysing independent cell subsets. Therefore, we recoded our MFI phenotype of interest (computed in CD4^+^ memory T cells) by computing, for each sample, the ratio of MFIs between CD4^+^ memory T cells and total CD4^+^ T cells. The advantage of this approach is the use of an internal control within the same sample, therefore providing control for technical variability. The drawback is the reliance on this additional phenotype to be biologically stable. This situation is similar to a gene expression analysis where a single gene is used for normalising the expression intensities; the underlying assumption is that the expression of this normalising gene is stable. In the example provided here the repeatability of the resulting phenotype was poor (*R* ^2^ = 0.37, Figure 5(b)), indicating that the repeatability of the MFI in the total CD4^+^ T cells is lower than what we observed in the CD4^+^ memory T cells.

## 3. Discussion

We have identified CD25 cell surface expression on CD4^+^ memory T cells to be a biologically stable phenotype, quantifiable by flow cytometry analysis. We have shown that the use of broad-spectrum fluorescent normalising beads significantly reduces the day-to-day variability of flow cytometry measurements. This normalisation could not have been achieved with the sole use of an isotype control, thus motivating the development of efficient tools for flow cytometry data normalisation.

We also investigated two alternative normalisation methods, less effective than normalising beads in this example but useful in situations where fluorescent beads are absent. A potentially useful approach consists of using the MFIs obtained from a different population of cells within the same sample, thus providing an internal normalisation. However, this procedure will only be useful in a situation where a different population with repeatable MFI values exists.

In spite of these results, normalisation of fluorescence intensity data from flow cytometry remains challenging. Indeed, controlling the technical variability of such a complex experimental procedure over extended periods of time is difficult. The development of methods for higher throughput flow cytometry, enabling the analysis of dozens of samples on the same day, may address some of these issues by shortening the duration of the experiment. However, when the phenotype of interest requires the analysis of fresh blood, which is the case in this study, the limiting factor becomes the number of blood samples collected per day, which is unlikely to become much higher. We have shown recently that CD25 cell surface expression on memory cells is decreased and more variable if frozen peripheral blood mononuclear cells are analysed [8], thereby ruling out storage of frozen cells as a way to increase throughput. Therefore, for such experiments the requirement for proper normalisation of flow cytometry data across several months remains a necessity.

An elegant approach to circumvent normalisation issues is the use of a nested design comparing, on each experimental day, both categories of samples (e.g., individuals with different genotypes, or cases/controls). When using this design, only phenotypes of individuals analysed on the same day are compared with each other, thus avoiding biases associated with day-to-day technical variability. When the study is balanced (i.e., the same number of samples from each category is analysed on each day) the loss of statistical power to detect phenotype differences is minimal, while the design becomes much more robust to technical variability.

## 4. Methods

### 4.1. Antibodies and Whole Blood Immunostaining

The anti-human monoclonal antibodies used for cell surface immunostaining were APC-conjugated anti-CD25 (BD Biosciences, clones M-A251 and 2A3), Alexa-Fluor 700-conjugated anti-CD4, Alexa-Fluor 488-conjugated anti-CD127, and Pacific Blue-conjugated anti-CD45RA (BioLegend). The isotype control antibodies used were APC-conjugated mouse IgG1 (BD Biosciences) and Alexa-Fluor 488-conjugated mouse IgG1 (BioLegend). To minimize potential variation due to antibody batch differences, all antibodies were obtained prior to the start of the experiment and all vials of antibody derived from the same clone and labelled with the same fluorochrome were pooled prior to usage. To better visualize lower-level CD25 expression, we increased CD25 detection sensitivity by simultaneously using two anti-CD25 monoclonal antibodies, (labelled with the same fluorochrome (clones 2A3 and M-A251), that recognize distinct epitopes on the CD25 molecule and therefore do not cross-compete. Prior to staining, whole blood samples were blocked with mouse IgG immunoglobulin (Sigma-Aldrich Company) at a concentration of 2 *μ*g per 100 *μ*L blood. All samples were stained within 5 hours postvenesection. After blocking, samples were stained for 40 minutes and then lysed for 10 minutes with freshly prepared 1X BD FACS Lysing Solution (BD Biosciences). Following erythrocyte lysis, samples were incubated at 4°C and were washed with BD CellWASH (BD Biosciences). The samples were fixed with freshly prepared 1X BD CellFIX (BD Biosciences). The samples were stored at 4°C until analysis by flow cytometry.

### 4.2. Flow Cytometry Analysis

All immunostained samples were analyzed using a BD LSRII Flow Cytometer with BD FACSDiVa Software (BD Biosciences). Each day donor samples were evaluated, we also analysed six peak normalising fluorospheres (Blank Beads and Calibration Beads, Dakocytomation) for MFI normalisation purposes. For our second dataset, where voltage settings were allowed to vary, six peak normalising fluorospheres (Blank Beads and Calibration Beads, Dakocytomation) and BD Calibrite APC Beads (BD Biosciences) were tested on each experimental day.

### 4.3. Data Processing and Statistical Analysis

The flow cytometry data were analyzed using FlowJo (Tree Star, Inc.). The remaining data processing/statistical analysis was performed using the R programing language. Gates were automatically extracted from the FlowJo output using an in-house XML parsing script based on the R XML 2.3.0 library. These gates were applied to the raw FCS files using the R flowCore 1.8.3 library. Repeatability *R* ^2^ values are estimated using [var(*X*) − ∑_*i*_(*X*_*i*_^1^ − *X*_*i*_^2^)^2^]/var(*X*) where (*X*_*i*_^1^)_*i* = 1_^*n*^ and (*X*_*i*_^2^)_*i* = 1_^*n*^ designate the first and second sets of replicates (*n* = 15 in this study).

## Supplementary Material

The supplementary figure shows a representative example of the flow cytometric gating used to define the CD4^+^ T cell subsets described in the main manuscript.
