# Supplementary material for: Fluorescence Intensity Normalisation: Correcting for Time Effects in Large-Scale Flow Cytometric Analysis
Source: Adv Bioinformatics. 2009 Nov 17;2009:476106. doi: 10.1155/2009/476106 (PMC2798117; doi:10.1155/2009/476106)
Supplement: Supplementary file 1 — The supplementary figure shows a representative example of the flow cytometric gating used to define the CD4+ T cell subsets described in the main manuscript. [file 476106.f1.pdf]

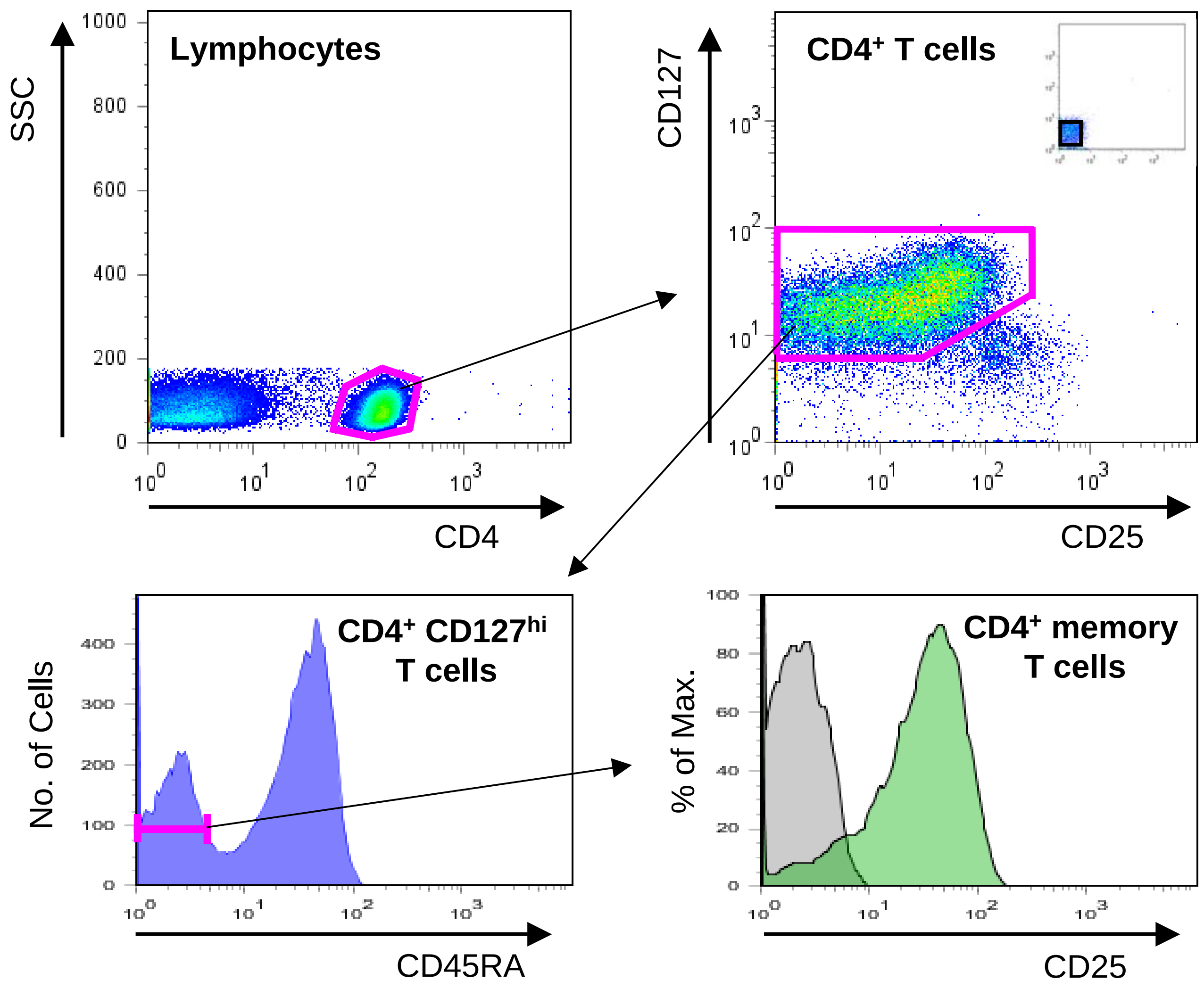

Supplementary Figure S1 Representative gating of total CD4$^+$ T cells and CD4$^+$ memory T cells. Lymphocytes were gated on their forward and side scatter properties (data not shown), and CD4$^+$ T cells were gated from the lymphocyte subset. The CD4$^+$ T cells were then gated on their IL-7R/CD127 expression (with the CD127$^{int-low}$ CD25^{hi} cells constituting the CD4$^+$ CD25^{hi} regulatory T cell subset). The inset plot shows isotype control staining. The CD127^{hi} cells were further gated on CD45RA to define the CD4$^+$ CD127^{hi} CD45RA^{neg} subset that is the CD4$^+$ memory T cell population. The mean fluorescence intensity of CD25 was measured for this CD4$^+$ memory T cell population (the isotype control staining is shown in grey). "% of Max" is calculated as the cell number in each bin divided by the cell number in the bin containing the largest cell number; this statistic is calculated as a normalisation for different numbers of events collected for each sample that is overlaid (http://www.treestar.com/flowjo/). SSC = Side scatter.
